# Interpreting dynamic-compression experiments to uncover the time dependence of freezing: Application to gallium

**DOI:** 10.1073/pnas.2424703122

**Published:** 2025-05-14

**Authors:** Jonathan L. Belof, Justin L. Brown, Philip C. Myint, Dane M. Sterbentz, Yue Hao, Brian S. Stoltzfus, Lorin X. Benedict

**Affiliations:** ^a^Lawrence Livermore National Laboratory, Livermore, CA 94550; ^b^Sandia National Laboratories, Albuquerque, NM 87185

**Keywords:** kinetics, shock, ramp, solidification, gallium

## Abstract

Using pulsed-power magnetic field sources to compress gallium to gigapascal pressures on nanosecond timescales, we report here experiments on shockless dynamic compression of a liquid metal. Time-resolved velocimetry data reveal signatures of rapid freezing from a metastable liquid state, and we demonstrate that the kinetics of this nonequilibrium solidification can be accurately simulated with a computational modeling framework we have developed in previous studies, where classical nucleation theory is coupled with hydrodynamics. Notably, velocity traces in some of our experiments show evidence of a phase transition, while others do not, even though other types of evidence suggest that solidification may be occurring in all of them. We explain how predictions made by our models regarding the presence or absence of these phase-transition signatures motivated additional experiments that later confirmed the theoretical predictions. Our analysis shows that due to the rapid, quasi-isentropic nature of the loading path, our experiments were able to compress liquid gallium to metastable states that are undercooled below the equilibrium melt temperature by more than 300 K and exhibit pressures that approach five times the equilibrium melt pressure. The understanding gained in this study should form the basis for future dynamic-compression experiments aimed at interrogating melt curves at high pressures.

## Significance

We present experiments in which liquid gallium is compressed to 0.3 million times atmospheric pressure within a few hundred nanoseconds. Because of this rapid compression, the gallium sample continues to remain as a metastable liquid at conditions far beyond those expected from the phase diagram of gallium, before it eventually solidifies on the nanosecond timescale of the experiments. We have analyzed our data with computational simulations, and predictions made by our simulations regarding certain experimental signatures motivated additional experiments that later confirmed these predictions. This demonstrates a more accurate diagnosis of delayed solidification in dynamically driven liquids, paving the way for future studies of rapid solidification and comprehensive analyses of experiments aimed at determining equilibrium melt temperatures in extreme conditions.

The high pressure melt temperature of materials is of great interest in planetary science and inertial fusion studies, because it delineates the thermodynamic conditions where materials can convect (in the molten regions of planets, and at interfaces between outer and inner layers of a fusion capsule). At sufficiently high pressures (e.g., multi-Mbars), dynamic compression (involving shocks, etc.) is the only practical means of reaching the desired conditions in a laboratory. Yet this poses a problem: If the pressure pulses are sufficiently rapid, it is not guaranteed that the material will have time to reach thermodynamic equilibrium during the duration of the experiment. Thus, issues of phase metastability and kinetics are a necessary consideration when high-pressure melt curves are inferred from dynamic measurements (1).

Because freezing is a transition from a disordered (liquid) state to an ordered (ideally, crystalline solid) state, it is a process that can be kinetically inhibited for short times. Thus, dynamically compressing a liquid into the (equilibrium) stability field of the solid often results in a supercooled state, where the thermodynamic conditions are such that the material would solidify if given enough time, but remains liquid during the duration of a short-time experiment. If the amount of undercooling is substantial, the detailed kinetic processes of eventual rapid solidification can then be quite different from those exhibited in slower crystal growth from melt (2).

Dynamically compressing from a liquid to a solid requires shockless or so-called ramp compression (since shocks heat the material enough to cause it to remain liquid even when compressed strongly), producing states that are closer to an isentrope than to a shock Hugoniot. Recent work has demonstrated eventual solidification at high pressures by ramp compressing a dense liquid which was initially created by shocking a solid (1, 3). In these studies, velocimetry (measuring the mechanical response of the compressed material) is used to diagnose the liquid ⟶ solid transition, along with (in ref. 1) time-resolved in situ X-ray diffraction. Inferred equivalent cooling rates of ∼10^9^ K/s can be achieved for high pressure liquids in this manner; these are nearly as high as the highest ambient pressure cooling rates observed in, e.g., splat cooling (4).

While time-resolved X-ray diffraction affords the most direct inference of the presence or absence of crystalline order (5), it is still very difficult to employ in the fastest dynamic-compression experiments (6), so velocimetry remains an essential diagnostic. The key signature of a phase transition in a velocimetry trace is a “loop feature” (discussed at length below) resulting from the density change from initial to final phases. However, as we will demonstrate, the absence of a clear loop feature does not necessarily imply the absence of a phase transition. For this reason, it is essential to employ continuum simulations when interpreting these measurements, where the material models used therein respect the multiphase, time-dependent nature of the transforming material. This is the approach we take here.

Over the past two decades, a growing body of experimental work has emerged on the kinetics of solidification in water under dynamic, quasi-isentropic compression (7–16). The particular solid phase that is formed in these experiments is thought to be ice VII. We have developed a general modeling framework (1, 15, 17–24), centered around our phase transformation kinetics code Samsa, that encapsulates the observations and understanding of the nucleation and growth of solid clusters provided by the experiments. With this approach, we found a scaling law which predicts the onset of solidification in greatly undercooled liquids, enabling the extraction of the equilibrium melt line from the analysis of a suitable collection of dynamic experiments (24).

In this work, we employ Samsa—in which we have implemented equations of state, interfacial free-energy models, and phase-transition kinetics models based on classical nucleation theory (CNT) and growth (25–27)—in concert with the Ares multiphysics hydrodynamics code (28–30) to simulate ramp compression of a preheated, melted Ga sample. We show that our modeling framework, assuming homogeneous nucleation of these deeply undercooled liquid states, is able to successfully describe our experiments on Ga, providing predictions on the presence/absence of solidification signatures (loop features) that motivated a set of follow-on experiments that later confirmed the theoretical predictions. With this understanding, we demonstrate that our combined theory + simulation + experimental approach is a crucial addition to the investigation of kinetically inhibited freezing at high pressures, needed for the eventual elucidation of equilibrium melt curves (24) in planetary science and related applications (1).

## Results

**Fig. 1.**
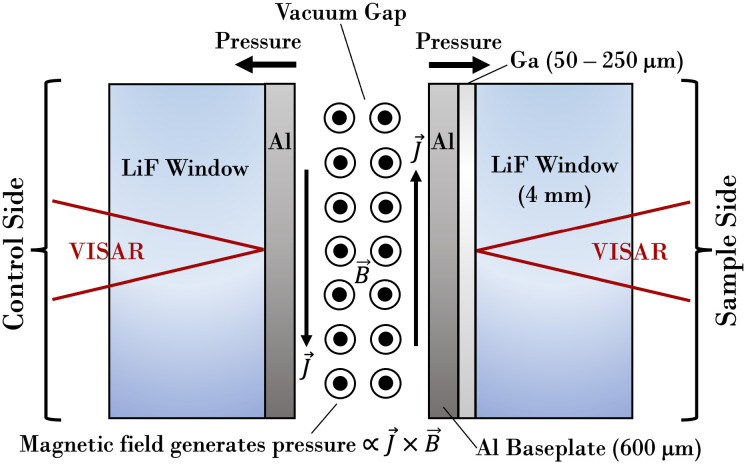
Illustration of our experimental setup, which is driven by a current pulse that is sent into a short-circuit load consisting of parallel aluminum (Al) electrodes, which contain type-II anodized coating (MIL-A-8625) on the surfaces in contact with Ga. This coating is essential to prevent Ga embrittlement (24) of the Al panels, which caused failures in preliminary testing of noncoated panels. The coatings are estimated to be ∼20 μm thick and are not believed to alter the interpretation of the data. The current pulse generates a magnetic pressure between the electrodes that, through the Lorentz force, generates a time-dependent stress wave on the inner surface of the electrodes. The stress wave then propagates through the Al electrode and into a transparent lithium fluoride (LiF) crystal on one side of the load (*Left* side) and through a thin liquid Ga cell also backed by LiF on the other side (*Right* side). The setup is compressed along a quasi-isentropic path (Fig. 2) as a result of this magnetically driven stress loading. The state of the system during the compression is monitored through Velocity Interferometer System for Any Reflector (VISAR) (32) measurements.

Experiments utilized the Thor pulsed-power machine (31) to compress liquid Ga cells to pressures of ∼20 to 30 GPa over timescales of several hundred nanoseconds. A schematic of the experimental configuration is shown in Fig. 1, and details of the experiment are described further in the figure caption. The entire setup is initially heated to a steady-state temperature of ∼308 K to maintain Ga in a liquid state prior to introducing the current pulse, and the pulse is tuned to give a desired magnitude and duration of the stress wave. The stress wave compresses both sides of the setup along the same quasi-isentropic path, and the initially liquid Ga sample continues to remain as a metastable liquid for some period of time along this path until it eventually solidifies. LiF was chosen for the window material because it has an excellent shock impedance match to Al, which minimizes pressure gradients through the Ga sample while simultaneously enabling optical diagnostics. The only experimental diagnostic consists of VISAR (32) velocimetry, and it is used to measure the velocity of the LiF interface on either side of the load. The velocity of the Al–LiF interface can be used to quantify the magnetic field boundary condition (33), which then enables Samsa/Ares simulations of the Ga side of the experiment. A more complete description of the experiments and boundary condition determination can be found in supplementary material of our recent study (24, 34).

**Fig. 2.**
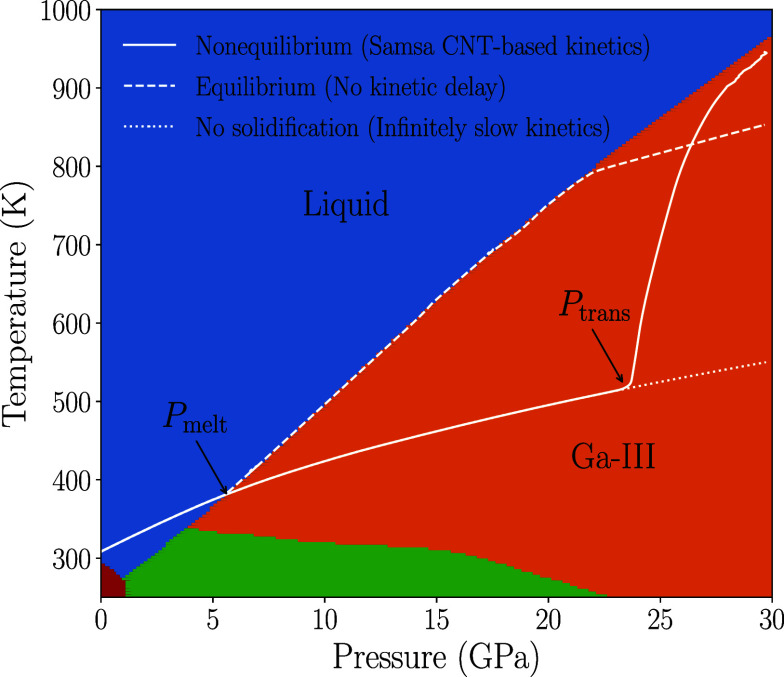
Loading paths under different kinetic scenarios (white curves) superimposed on the phase diagram of Ga from a recent multiphase EOS (35) for that material. In all of our experiments and simulations, a Ga sample at ambient pressure ($10^{-4}$ GPa) is preheated to 306 to 309 K so that it melts and enters the liquid region of the phase diagram (blue), after which it is then ramp compressed quasi-isentropically toward the Ga-III solid region (orange). The Ga-I (red) and Ga-II (green) solid phases are not accessed in this study but are shown here for completeness. The three different white curves are taken from simulations of one of our experiments (Shot 2) performed with our Samsa code (19, 22), which features phase-transition kinetics models based on classical nucleation theory (CNT) (25–27). The dotted curve shows the trajectory if solidification to Ga-III was suppressed so that the Ga sample continues to indefinitely follow the liquid-phase quasi-isentrope. The dashed curve shows the opposite scenario if the transition to Ga-III were to occur in an equilibrium manner as soon as the quasi-isentrope intersects the melt curve at $P_{\mathrm{melt}}$. The solid curve corresponds to the situation that actually occurs experimentally; it depicts an intermediate scenario where the Ga continues to exist as a nonequilibrium, metastable liquid for some period of time, and the transition to Ga-III becomes macroscopically observable only after achieving a pressure $P_{\mathrm{trans}}$ that lies well past $P_{\mathrm{melt}}$.

Fig. 2 illustrates the temperature–pressure loading path in our Samsa/Ares simulations (36). Of the three scenarios (white curves) depicted in the figure, the most interesting case—which is also the one that is borne out experimentally—corresponds to the solid white curve, in which Ga continues to exist as a nonequilibrium, metastable liquid until it achieves a pressure $P_{\mathrm{trans}}$ that lies well past the equilibrium melt pressure $P_{\mathrm{melt}}$. At $P_{\mathrm{trans}}$, the liquid is undercooled by over 300 K below its equilibrium melt temperature, resulting in a very strong driving force for solidification that causes the initially liquid Ga to solidify to the Ga-III phase on the nanosecond timescales of the experiments.

**Fig. 3.**
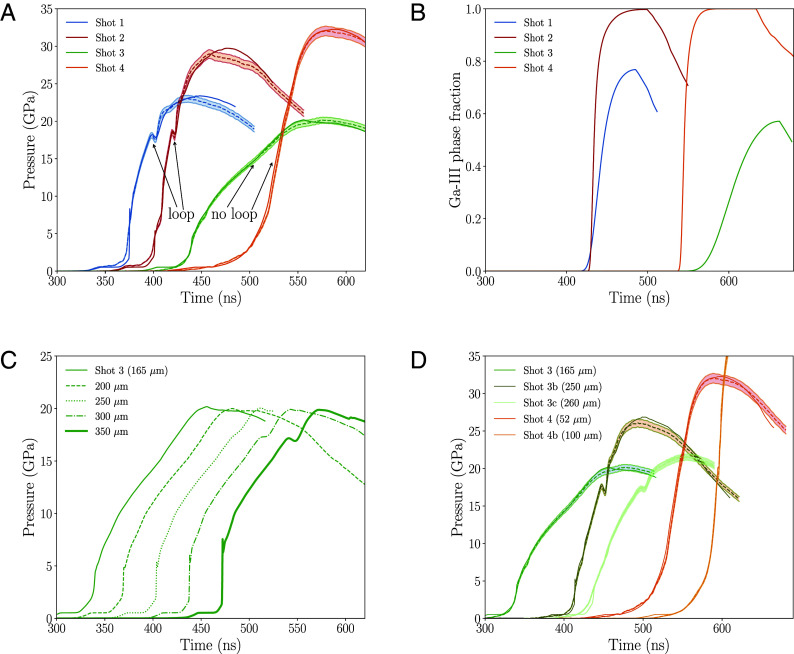
(*A*) Comparison of velocimetry profiles at the Ga–LiF window interface in our Samsa/Ares simulations (solid curves) vs. our experiments (dashed curves) in four of our shots. Two of the shots exhibit a loop feature that is signature of the phase transition from liquid to Ga-III; (*B*) phase fraction of Ga-III vs. time in our simulations for all four shots; (*C*) simulations of Shot 3, in which the thickness of the Ga sample is estimated to be 165 μm, and alternative versions of that shot where the thickness of the Ga target has been increased to explore the effects of sample thickness; (*D*) simulations (solid curves) and experiments (dashed curves) for Shots 3b and 3c, which are colored in different shades of green like Shot 3, as well as results for Shot 4b, which is colored in a different shade of orange from Shot 4. In all panels, the time origin of the wave profiles have been shifted by arbitrary amounts for clarity. The shaded regions enveloping the experimental dashed curves in (*A* and *D*) denote ±2% error bars.

Velocimetry wave profiles collected through techniques like VISAR (32) are the most widely used and well-known source of data for analyzing phase-transition kinetics. Fig. 3*A* presents wave profiles for four of our experiments, here labeled Shots 1, 2, 3, and 4 (these are a subset of 14 shock + ramp experiments we performed on Ga). The most salient feature in the wave profiles is the loop signature that appears in two of the experiments, Shots 1 and 2. The loop is an indication of the phase transition from liquid to Ga-III, and the top of this loop roughly corresponds to $P_{\mathrm{trans}}$ illustrated in Fig. 2. There is a momentary drop in pressure because the newly formed Ga-III is denser than the liquid phase. As stated in *Materials and Methods*, our models involve only one adjustable parameter, which we denote as ξ and which pertains to some aspect of the interfacial free energy σ between the liquid and Ga-III phases (see our *Materials and Methods*, as well as ref. 23, for its description); according to CNT, σ strongly influences the solidification kinetics because the nucleation rate of solid clusters is proportional to $\text{exp}(-\sigma^{3})$. We have found that by setting ξ=0.665, our simulations accurately reproduce the timing of the loop feature in our experiments, indicating that the kinetics of the phase transition is accurately captured by the simulations. Interestingly, both the experimental and simulation results for Shots 3 and 4 do not exhibit a loop feature, suggesting that perhaps a phase transition did not occur in these particular shots. However, the time-dependent phase fractions of Fig. 3*B* show that, at least in the simulations, solidification to Ga-III occurred in all of the shots, even in the two where the loop signature is absent. Indeed, one might expect that if solidification to Ga-III occurs in one of the experiments, it should occur in all of the other experiments as well since they all access similar temperature–pressure conditions. Similar observations were reported in a recent study on water (37), where diffraction measurements confirmed the occurrence of solidification even in experiments where the loop feature was not present. This raises a puzzling question: Why does the loop appear in the velocimetry traces in some experiments but not in others even though a phase transition may have occurred in all of them?

Fig. 3*C* provides some insight into this question. It shows how simulated wave profiles of Shot 3, which is one of the two experiments that did not exhibit a loop, would look if the thickness of Ga sample were increased beyond its estimated value of 165 μm but all other aspects of the experiment remain unchanged. The figure demonstrates that the loop feature is recovered if the Ga sample is sufficiently thick. This finding from the simulations motivated us to perform follow-on experiments in which we increased the sample thickness. These additional experiments, which one can think of as variations on Shots 3 and 4 with thicker samples (but with other features as similar as possible), are illustrated in Fig. 3*D*. We have simulated these later experiments with the same common model (i.e., ξ=0.665) that we have used for simulating all of the earlier experiments in Fig. 3*A*. As predicted by the simulations, the loop feature appears in the variations involving thicker samples.

## Discussion

**Fig. 4.**
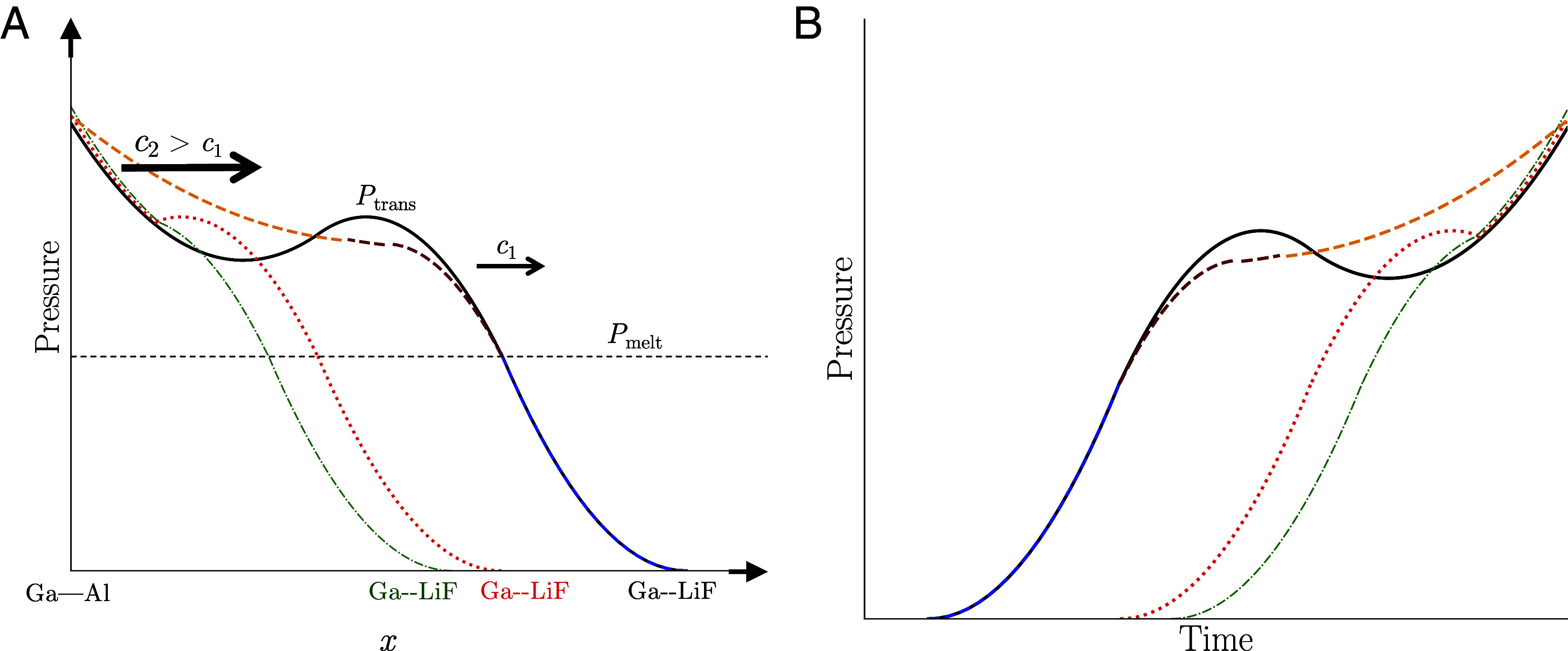
(*A*) Snapshot of the pressure P as a function of position x in the Ga sample as a result of quasi-isentropic compression under both nonequilibrium (black solid curve) and equilibrium (blue–brown–orange dashed curve) scenarios, where the different segments of the latter curve correspond to liquid (blue), mixed phase (brown), and Ga-III (orange). The red dotted and green dashed–dotted curves depict nonequilibrium scenarios for thinner samples. As illustrated in Fig. 1, the pressure waves propagate from the Ga–Al interface toward the Ga–LiF interface. The waves are nonlinear in nature so that their propagation velocity c increases with pressure, resulting in the wave velocity $c_{2}$ near the Ga–Al interface being greater than the wave velocity $c_{1}$ closer to the Ga–LiF interface. This wave dispersion suppresses the loop signature of the phase transition. (*B*) The corresponding pressure vs. time wave profiles at the Ga–LiF interface, which is the location in the experimental setup where the VISAR data are collected.

Figs. 2 and 4 together illustrate the reason for this dependence of the wave profiles on the thickness of the Ga samples. Fig. 4*A* sketches a snapshot of pressure vs. position x in the sample at a fixed instance in time. The blue–brown–orange curve represents the pressure profile if the solidification were to happen in a purely equilibrium manner. The same equilibrium loading path in temperature–pressure space is represented by the white dashed curve in Fig. 2. The sample starts at ambient pressure in the liquid phase (colored in blue in Fig. 4). As it gets compressed, it remains purely as a liquid until the quasi-isentrope intersects with the melt curve at a pressure $P_{\mathrm{melt}}$ and enters the mixed-phase region (brown). As the Ga gets compressed deeper into the mixed-phase region, it rides along the melt curve in temperature–pressure space, again referring to the white dashed curve in Fig. 2. In the course of doing so, more and more of the solid forms at the expense of the liquid, until eventually the Ga becomes completely solid and is subsequently compressed along the Ga-III branch of the quasi-isentrope (orange in Fig. 4). In reality, however, with the presence of kinetics the pressure profile more closely resembles the black curve in Fig. 4. Instead of solidifying at $P_{\mathrm{melt}}$, the Ga continues to follow the liquid branch of the quasi-isentrope until it achieves a pressure $P_{\mathrm{trans}}$, where the liquid is so far beyond its stability field (see the solid white curve in Fig. 2) that the kinetics of the phase transition becomes comparable to the timescale of the experiment itself so that solidification becomes macroscopically observable. There is a subsequent decrease in pressure as the Ga heads back toward the melt curve as it solidifies. However, this decrease in pressure is only momentary, as pressure waves from the regions of Ga closer to the Ga–Al interface, which are at a higher pressure and a more mature stage in the solidification process, propagate toward the Ga–LiF interface. This momentary drop followed by a subsequent increase in pressure from the arrival of the higher-pressure waves is the underlying reason for the presence of the phase-transition loop signature in the wave profiles, as shown in Fig. 4*B*.

But what about the absence of this signature in certain experiments? This is due to the nonlinear, dispersive nature of these waves, which causes the propagation velocity to increase with pressure. If the Ga sample were sufficiently thin, or alternatively, if the compression rate of the experiment were sufficiently high, the faster waves emanating from the higher-pressure regions of the sample would overtake the slower, lower-pressure waves before these lower-pressure waves arrive at the Ga–LiF interface, which is the location in the Ga sample where the velocimetry is being recorded. This would suppress the loop signature in the wave profiles, resulting instead in a partially formed loop (e.g., the red curve in Fig. 4*B* or even a continually increasing pressure profile in which the loop feature is entirely absent, like those shown for Shots 3 and 4 in Fig. 3*A* or the green curve in Fig. 4*B*, despite the fact that solidification might nonetheless be occurring in these experiments. As a cautionary aside, we note that although the loop signature requires a sufficiently thick sample, an overly thick sample would result in shock formation—again due to the nonlinear nature of the waves—which would not only suppress the signature, but likely even the solidification process itself since the high-temperature and entropy states that result from shock formation inhibit solidification.

In order to quantify these arguments, we turn to Table 1. This table compares two different length scales: the thickness $L_{\mathrm{Ga}}$ of the Ga sample and the product $\Delta t\cdot c_{S}$ defined in the table caption. The latter is a length scale that characterizes the phase-transition-plus-wave-dispersion process illustrated in Fig. 4. If the Ga sample is too thin so that $L_{\mathrm{Ga}}<\Delta t\cdot c_{S}$, the faster/higher-pressure waves arriving from the Ga–Al interface will be able to overtake the slower/lower-pressure waves near the Ga–LiF interface so that the VISAR signal at the Ga–LiF interface will be unable to detect the drop in pressure, leading to the absence of the loop feature. The loop is observed only in shots with sufficiently thick samples (correlated with shots in which $L_{\mathrm{Ga}}>\Delta t\cdot c_{S}$) where this overtaking process does not occur before the VISAR is able to detect and record the loop signature.

**Table 1.** Comparison of the product $\Delta t\cdot c_{S}$ vs. the thickness $L_{\mathrm{Ga}}$ of the Ga sample

**Shot**	**Δ*t* (ns)**	**Δ*t* · *c_S_* (μm)**	***L_Ga_* (μm**)	**Δ*t* · *c_S_* < *L_Ga_*?**	**Loop?**
1	45.9	160	195	Yes	Yes
2	41.9	146	150	Yes	Yes
3	56.1	194	165	No	No
3b	62.0	214	250	Yes	Yes
3c	53.7	186	260	Yes	Yes
4	52.3	181	52	No	No
4b	13.8	48	100	Yes	Yes

Here, $c_{S}$ is the liquid sound speed evaluated at $\left(T_{\mathrm{melt}},P_{\mathrm{melt}}\right)$, and Δt represents the time duration of the phase transition and is defined as $\Delta t=t_{\phi \;\text{max}}-t_{\mathrm{initiation}}$, where $t_{\phi \;\text{max}}$ is the time corresponding to when the solid phase fraction ϕ reaches its maximum value (this maximum value =1 if the phase transition reaches completion in the simulation), and $t_{\mathrm{initiation}}$ is the time when the nucleation rate achieves its maximum value. $t_{\mathrm{initiation}}$ occurs slightly before $P_{\mathrm{trans}}$ is achieved, in the very early stages of the phase transition where the system is almost entirely composed of metastable liquid (ϕ<0.001) and the free-energy difference between liquid and solid that drives the phase transition is greatest.

Through a series of experiments and computational simulations, we have investigated the kinetics of solidification in gallium under rapid, quasi-isentropic compression. The experiments are able to achieve metastable liquid states that are undercooled by hundreds of Kelvin and many times past the equilibrium melt pressure. Although X-ray diffraction offers a powerful means to visualize phase-transition kinetics under dynamic compression, obtaining reliable quantitative assessments of phase fractions from diffraction remains an ongoing challenge, and as a result, VISAR wave profiles continue to be the most commonly employed experimental diagnostic for phase-transition kinetics. It has long been recognized that solidification can manifest in a distinct loop feature in the wave profiles, yet this feature was absent in some of our experiments, even though our simulations suggest that solidification had occurred in them. Using arguments based on the dispersive nature of the waves in which their speed increases with pressure, we have devised an explanation for why—even if solidification occurs—the sample must be sufficiently thick and/or the compression rate must be sufficiently low in order for the velocimetry trace to exhibit a loop feature. Motivated by this theory, we performed a follow-on set of experiments with thicker samples where the loop signature was indeed recovered. Additional insight could be gained if in situ optical pyrometry (38) (or some other temperature diagnostic) is fielded in such experiments, since the onset of solidification is predicted to be accompanied by a sharp rise in T (Fig. 2). As more diagnostics are brought to bear on greatly undercooled liquid Ga at high pressures, it may then be possible to explore novel structural motifs recently discovered within its equilibrium stability field (39).

With this experiment + theory + simulation approach, we are now able to design future experiments to best constrain our remaining uncertainties in materials-specific time-dependent solidification models. This will help us to elucidate the amount of undercooling/overdriving that occurs in a given experiment. In addition to furthering the study of phase transitions in extreme conditions generally, this will provide crucial guidance in studies motivated by planetary science, where the equilibrium high-pressure melt temperature (24) is the desired quantity (as in, e.g., ref. 1).

## Materials and Methods

In this section, we provide a brief summary of our computational modeling framework. As stated in the main text above, we run two codes together: the kinetics code Samsa (19, 22) and the multiphysics hydrodynamics code Ares (28–30). Details about the models we have implemented in Samsa and the coupling between these two codes are provided in our paper from 2020 (22) and in supplemental material accompanying our 2018 paper (19). Here, for the purposes of making the current study more self-contained, we focus primarily on some of the aspects relevant to the simulations of our Ga solidification experiments.

**Table 2.** Same as Table 1, except featuring Samsa/Ares simulations that were run with a different EOS and interfacial free-energy model, as described at the end of *Materials and Methods*

**Shot**	**Δ*t* (ns)**	**Δ*t* · *c_S_* (μm)**	***L_Ga_* (μm)**	**Δ*t* · *c_S_* < *L_Ga_*?**	**Loop?**
1	32.1	105	195	Yes	Yes
2	15.4	50	150	Yes	Yes
3	60.6	195	165	No	No
3b	25.9	84	250	Yes	Yes
3c	76.7	248	260	Yes	Yes
4	17.4	56	52	No	No
4b	6.2	21	100	Yes	Yes

We represent our experiments in Ares with a one-dimensional finite-element mesh on which the governing conservation equations are solved through an arbitrary Lagrangian–Eulerian framework. The setup depicted in Fig. 1 is discretized with a dense mesh composed of hundreds of points, with an initial grid spacing of 5 μm in the Al and LiF regions and 0.5 μm in the Ga region. Time-step control is determined through the capillary-length-based scheme described in ref. 19, with maximum and minimum step sizes of 0.1 ns and 1 fs, respectively. We represent the magnetic drive as a time-dependent pressure source that acts on the interface between Al and the vacuum gap (Fig. 1). This time-dependent drive measurement is determined through the least-squares procedure described in earlier publications (24, 40, 41) and is read in as an input file in our simulations.

At each time step, Ares numerically integrates the governing equations describing the hydrodynamics to determine the energy E, density ρ, and solid phase fraction ϕ in each mesh point at the next time step. This (E,ρ,ϕ) is used by Samsa, which effectively serves as a library that gets called by Ares, to perform phase-behavior computations (22) to determine the corresponding temperature T and pressure P, which Samsa then feeds into its phase-transition-kinetics models to calculate the time derivative ∂ϕ/∂t of the phase fraction. This (T,P,∂ϕ/∂t) is in turn utilized by Ares to determine (E,ρ,ϕ) at the next time step, which begins the new cycle. The phase-behavior computations in Samsa allow for the possibility of different phases of a material to be at different pressures through the presence of Laplace pressure, and it also allows for thermal disequilibria so that phases remain at different temperatures, the latter of which we employed in our previous work on water (15, 19, 23). For our Ga simulations, however, we neglect Laplace pressure and use a single-temperature model so that the liquid and solid phases are always in thermal equilibrium. Samsa computes ∂ϕ/∂t through the Kolmogorov–Johnson–Mehl–Avrami equation (22, 42–45), which relates ∂ϕ/∂t to the current value of ϕ and time integrals of the nucleation rate J of solid clusters and the growth rate γ of these clusters. We model J in accordance with CNT (25–27), which states that J is proportional to $\text{exp}(-\Delta G^{*}/k_{B}T)$. Here, $k_{B}$ is the Boltzmann constant, and the nucleation energy barrier $\Delta G^{*}/k_{B}T=16\pi \sigma^{3}/\left[3k_{B}T{\left(\rho_{\mathrm{solid}}\Delta \mu \right)}^{2}\right]$, where σ is the solid–liquid interfacial free energy and Δμ is the chemical potential (bulk free energy) difference between the two phases. Finally, we calculate the growth rate γ with the expression given in earlier studies (2, 46): $\gamma ={\left(RT\right)}^{1/2}\left[1-\text{exp}\left(-\Delta \mu /k_{B}T\right)\right]$, in which R is the gas constant expressed on a per-mass basis.

The computations described in this section require the implementation of constitutive models into the codes, including models for equations of state, the solid–liquid interfacial free energy in Ga, and strength effects that arise from deviatoric stresses in solids. We do not prescribe a strength model for Ga, but for LiF, we use that presented by Ao et al. (47). We represent the EOS of Ga, Al, and LiF with the models developed by Wu et al. (35), Holian (48), and Davis et al. (7271v3) (49), respectively. We model the solid–liquid interfacial free energy σ in Ga with our formulation described in Sterbentz et al. (23), which represents the interface as a layer that is multiple atoms thick and is a modification of a single-atom-layer model developed earlier by Jian et al. (50). Our model in ref. 23 features an adjustable parameter ξ, which in a rough sense represents the strength of the average interatomic interactions within the interface relative to those in the bulk liquid and solid phases. This ξ is the only adjustable parameter that appears in our computational modeling framework, and all of the Ga simulations presented in this study were performed with a common model in which we have set ξ=0.665. While our hydro + solidification kinetics simulations explicitly model homogeneous nucleation (likely appropriate for our cases of deep undercooling, where all Ga atoms are potential nucleation sites), it is possible that this value reflects some effective additional (but relatively small) contribution from heterogeneous nucleation at the interfaces between Ga and neighboring materials (and/or impurities within bulk Ga). Table 2 presents results from an alternative set of simulations in which we have swapped out the Wu et al. EOS for Ga (35) with a different Ga EOS by Crockett and Greeff (51) and have additionally replaced our multilayer σ model with the earlier single-layer σ model by Jian et al. (50) We are able to achieve similar quantitative agreement with experimental wave profiles using these alternative models (including the presence or absence of the loop feature depicted in Figs. 3 and 4 and discussed in the accompanying text), which demonstrates that our conclusions regarding the phase-transition kinetics hold across at least these different choices of EOS and interfacial free energy models.

## Data Availability

Datasets generated during the current study have been deposited in GitHub (https://github.com/philipmyint/pnas_2025) (36).
